# Direct Position Determination of Non-Gaussian Sources for Multiple Nested Arrays: Discrete Fourier Transform and Taylor Compensation Algorithm

**DOI:** 10.3390/s24123801

**Published:** 2024-06-12

**Authors:** Hao Hu, Meng Yang, Qi Yuan, Mingyi You, Xinlei Shi, Yuxin Sun

**Affiliations:** 1 College of Electronic Information Engineering, Nanjing University of Aeronautics and Astronautics, Nanjing 211106, China; 18061368794@163.com (H.H.); yangmeng19861213@126.com (M.Y.); yuanqi@nuaa.edu.cn (Q.Y.); sunyuxin@nuaa.edu.cn (Y.S.); 2 Key Laboratory of Dynamic Cognitive System of Electromagnetic Spectrum Space, Nanjing University of Aeronautics and Astronautics, Ministry of Industry and Information Technology, Nanjing 211106, China; 3 Jiangsu Automation Research Institute, Lianyungang 222000, China; 4 China Electronics Technology Group Corporation No.36 Research Institute, Jiaxing 314033, China; youmingyi@126.com; 5 National Key Laboratory of Electromagnetic Space Security, Jiaxing 314033, China

**Keywords:** non-Gaussian signal, multiple nested arrays, direct position determination, Discrete Fourier Transform, Taylor compensation

## Abstract

This paper delves into the problem of direct position determination (DPD) for non-Gaussian sources. Existing DPD algorithms are hindered by their high computational complexity from exhaustive grid searches and a disregard for the received signal characteristics by multiple nested arrays (MNAs). To address these issues, the paper proposes a novel DPD algorithm for non-Gaussian sources with MNAs: the Discrete Fourier Transform (DFT) and Taylor compensation algorithm. Initially, the fourth-order cumulant matrix of the received signal is computed, and the vectorizing method is applied. Subsequently, a computationally efficient DPD cost function is proposed by leveraging a normalized DFT matrix to reduce complexity. Finally, first-order Taylor compensation is utilized to enhance the accuracy of the localization results. The superiority of the proposed algorithm is demonstrated through numerical simulation results.

## 1. Introduction

The advancement of wireless positioning technology has led to its widespread application in various sectors, including military defense, emergency rescue [1,2], resource exploration, intelligent transportation, and more [3,4,5]. Precisely locating enemy radiation sources is crucial for victory in battlefield scenarios. Wireless localization technology is categorized into traditional two-step positioning and direct position determination (DPD) technology [6,7,8].

The two-step positioning framework can be further classified into angle of arrival (AOA) [9], time of arrival (TOA) [10], time difference of arrival (TDOA) [11], received signal strength (RSS) [12], and other categories based on intermediate parameters estimation. While this technology estimates intermediate parameters from received signals and then solves spatial geometry problems to determine radiation source positions, it suffers from information loss between steps and suboptimal accuracy due to errors in parameter matching processes [13].

A DPD algorithm is suggested in [14] as a solution to address the challenges mentioned. This algorithm has garnered attention for its ability to achieve superior localization accuracy compared to the two-step algorithm, particularly in low signal-to-noise ratio (SNR) environments. The DPD algorithm directly determines the source position from the original received data, eliminating the need for estimating signal parameters and thereby avoiding estimation errors associated with intermediate parameters in the two-step approach. Moreover, the DPD algorithm does not require a matching procedure. While no intermediate parameters are essential in the DPD framework, certain signal parameters must still be taken into account in the algorithm model. The maximum likelihood (ML) DPD estimator in [15] incorporates joint information from angle of arrival (AOA) and time difference of arrival (TDOA) to achieve high localization accuracy through exhaustive search, albeit at the cost of increased complexity in scenarios with multiple sources. To mitigate this complexity, the subspace data fusion (SDF) technology [16] based on multiple signal classification (MUSIC) [17] is proposed. Furthermore, the Capon DPD algorithm, which avoids eigenvalue decomposition (EVD), is introduced for situations with multiple sources [18]. However, all the aforementioned algorithms treat the source signal as arbitrary, whereas studies have proved that better positioning accuracy can be achieved when the property of the source signal is a priori known [19,20]. A localization algorithm tailored for orthogonal frequency division multiplexing (OFDM) signals is detailed in [21]. Due to the high computational demands of ML algorithms, an extended SDF DPD algorithm is proposed in [22]. Additionally, features of non-circular signals are harnessed to enhance positioning accuracy and increase the degree of freedom (DOF) as evidenced by studies such as [23,24,25].

In the realm of localization research, it is commonly presumed that a signal adheres to a Gaussian distribution, with the second-order cumulant (SOC) [26] being utilized to derive a probability density function that encapsulates all signal information. However, practical scenarios often involve signals that deviate from Gaussian distribution, rendering lower-order cumulants insufficient in capturing all signal details. Therefore, the fourth-order cumulant (FOC) introduced in [27] is employed for signal analysis. Unlike SOC, FOC is adept at disregarding Gaussian noise and expanding array elements, leading to enhanced parameter estimation precision as evidenced in [28,29,30]. Moreover, prevalent position estimation techniques rely on uniform linear arrays (ULAs), which suffer from densely positioned elements, inducing heightened mutual coupling. Proposals to mitigate this challenge include sparse arrays with larger apertures and reduced mutual coupling, exemplified by classic coprime arrays and nested arrays (NAs) as put forth in [31,32,33].

In this paper, we propose a novel DPD algorithm utilizing multiple nested arrays (MNAs) for non-Gaussian sources. The main contributions are summarized as follows:

- The property of non-Gaussian sources is fully exploited to suppress Gaussian noise and augment the virtual array aperture, which benefits the available degrees of freedom (DOFs).
- We propose a novel low-complexity DPD algorithm of non-Gaussian sources utilizing MNAs. We deploy the Discrete Fourier Transform (DFT) method to construct a computationally efficient DPD cost function to reduce the high computational complexity caused by exhaustive grid search.
- We utilize the Taylor compensation method to improve the localization accuracy at the expense of calculating the position estimation bias. It should be emphasized that even when the source position does not fall on the preset grid, the proposed algorithm can still estimate the position of sources accurately.
- Complexity analysis and extensive numerical results are presented to verify the superiority of the proposed algorithm in terms of location accuracy, resolution capability, and computational complexity.

The following structure of the article is, the MNAs localization model is introduced in Section 2. The proposed algorithm is derived in Section 3. Performance analysis, numerical results, and conclusions are drawn in Section 4, Section 5 and Section 6, respectively.

*Notations:* Vectors and matrices are lower-case bold and upper-case bold, respectively. ${\left(\cdot \right)}^{T}$, ${\left(\cdot \right)}^{*}$, and ${\left(\cdot \right)}^{H}$ denote transposition, conjugate, and Hermitian transpose, respectively. $x\left(n\right)$ extracts the *n*th element of vector x; $E\left(\cdot \right)$ represents the expectation operator; ⊗ is the Kronecker product; $\mathrm{vec}\left(\cdot \right)$ stacks the columns of a matrix into a vector; $\partial \left(x\right)/\partial \left(y\right)$ denotes the derivation of *x* with respect to *y*; $\mathrm{diag}\left(x\right)$ turns vector x into a diagonal matrix; and ∥·∥ denotes the $l_{2}$ norm.

## 2. Model Formulation

**Figure 1:**
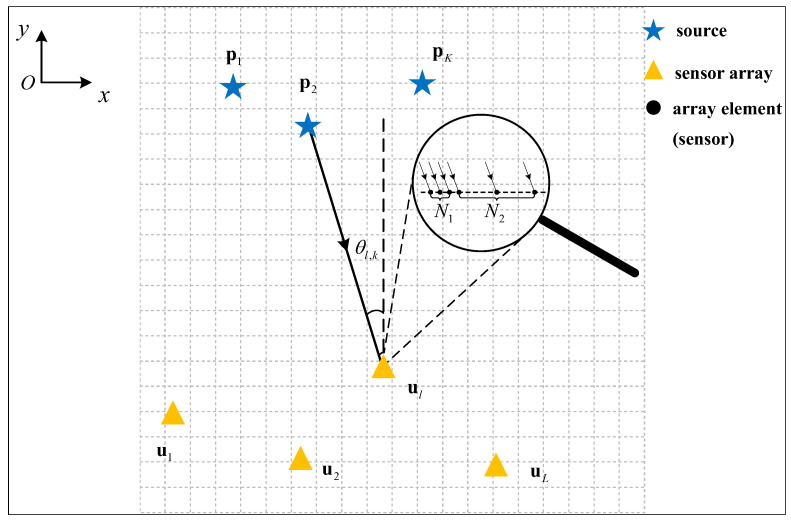
Geometry of multiple nested arrays localization.

In the context depicted in Figure 1, we analyze a scenario where *K* incoherent non-Gaussian sources emit narrow-band stationary signals in a two-dimensional localization setting, with unknown positions. It is assumed that the quantity of sources *K* is predetermined, and various established techniques can be employed to ascertain it [34,35]. To pinpoint these unidentified sources, we deploy *L* spatially distributed sensor arrays, each furnished with a NA comprising *M* array elements. The positions of the sources and sensor arrays are represented as $p_{k}={\left[x_{k},y_{k}\right]}^{T}\left(k=1,\cdots ,K\right)$ and $u_{l}={\left[x_{l},y_{l}\right]}^{T}\left(l=1,\cdots ,L\right)$, respectively. The signal received by the *l*th sensor array at the *t*th (t=1,⋯,T) snapshot can be formulated as per [36]:

(1) $$x_{l}\left(t\right)=A_{l}\left(p\right)s\left(t\right)+n_{l}\left(t\right)$$

with the following notational definitions:

- $p={\left[p_{1}^{T},\cdots ,p_{K}^{T}\right]}^{T}\in C^{2K\times 1}$ is the position vector of *K* sources;
- $A_{l}\left(p\right)=\left[a_{l}\left(p_{1}\right),a_{l}\left(p_{2}\right),\cdots ,a_{l}\left(p_{K}\right)\right]\in C^{M\times K}$ is the array manifold of the *l*th NA, and $a_{l}\left(p_{k}\right)={\left[e^{j2\pi d_{1}\frac{\sin\varphi_{l,k}}{\lambda }},e^{j2\pi d_{2}\frac{\sin\varphi_{l,k}}{\lambda }},\cdots ,e^{j2\pi d_{M}\frac{\sin\varphi_{l,k}}{\lambda }}\right]}^{T}\in C^{M\times 1}$ denotes the array steering vector, where $\sin\varphi_{l,k}=\frac{p_{k}\left(1\right)-u_{l}\left(1\right)}{∥u_{l}-p_{k}∥}$. Array element locations $d_{1},d_{2},\cdots ,d_{M}$ are given by the set
  (2) $$S=\left\{d,2d,\cdots ,N_{1}d,\left(N_{1}+1\right)d,2\left(N_{1}+1\right)d,\cdots ,N_{2}\left(N_{1}+1\right)d\right\}$$
  where $M=N_{1}+N_{2}$, and *d* denotes the space between array elements, which is usually set as half of wavelength λ/2;
- $s\left(t\right)={\left[s_{1}\left(t\right),s_{2}\left(t\right),\cdots ,s_{K}\left(t\right)\right]}^{T}\in C^{K\times 1}$ is the signal vector transmitted from the *k*th source at time *t*, where k=1,2,⋯,K, t=1,2,⋯,T;
- $n_{l}\left(t\right)$ denotes the independent additive white Gaussian noise vector of the *l*th sensor array.

## 3. Proposed Algorithm

Define the FOC of a stationary stochastic process *x* as [37]:

(3) $$Cum\left(x_{k_{1}},x_{k_{2}},x_{k_{3}}^{*},x_{k_{4}}^{*}\right)=E\left(x_{k_{1}}x_{k_{2}}x_{k_{3}}^{*}x_{k_{4}}^{*}\right)-E\left(x_{k_{1}}x_{k_{2}}\right)E\left(x_{k_{3}}^{*}x_{k_{4}}^{*}\right)-E\left(x_{k_{1}}x_{k_{3}}^{*}\right)E\left(x_{k_{2}}x_{k_{4}}^{*}\right)-E\left(x_{k_{1}}x_{k_{4}}^{*}\right)E\left(x_{k_{2}}x_{k_{3}}^{*}\right)$$

where $k_{1}$, $k_{2}$, $k_{3}$, $k_{4}$, $1\leq k_{1},k_{2},k_{3},k_{4}\leq T$ are integers. And the FOC matrix of the received signal $x_{l}\left(t\right)$ can be expressed as [38,39]:

(4) $$R_{l,4}=\sum_{k=1}^{K}c_{s_{k},4}\left[a_{l}\left(p_{k}\right)⊗a_{l}^{*}\left(p_{k}\right)\right]{\left[a_{l}\left(p_{k}\right)⊗a_{l}^{*}\left(p_{k}\right)\right]}^{H}=\sum_{k=1}^{K}c_{s_{k},4}a_{l,4x}\left(p_{k}\right)a_{l,4x}^{H}\left(p_{k}\right)$$

where $a_{l,4x}\left(p_{k}\right)=a_{l}\left(p_{k}\right)⊗a_{l}^{*}\left(p_{k}\right),1\leq k\leq K$, $c_{s_{k},4}=Cum\left(s_{k}\left(t\right),s_{k}\left(t\right),s_{k}^{*}\left(t\right),s_{l,k}^{*}\left(t\right)\right)$ denotes the FOC of the *k*th signal and is calculated using (3). Notably, the elements of $a_{l,4x}\left(p_{k}\right)$ can be constructed with $e^{j2\pi \left(d_{i}-d_{j}\right)\frac{\sin\varphi_{l,k}}{\lambda }}$, $d_{i},d_{j}\in S$. In practice, $a_{l,4x}\left(p_{k}\right)$ can be considered the steering vector of $R_{l,4}$, which expands from $a_{l}\left(p_{k}\right)$. To further improve the accuracy of localization, we vectorize $R_{l,4}$ by column [40]:

(5) $${\tilde{z}}_{l}=\mathrm{vec}\left(R_{l,4}\right)={\tilde{A}}_{l}\left(p\right)c$$

where ${\tilde{A}}_{l}\left(p\right)$ takes the form:

(6) $${\tilde{A}}_{l}\left(p\right)=\left[a_{l,4x}^{*}\left(p_{1}\right)⊗a_{l,4x}\left(p_{1}\right),\cdots ,a_{l,4x}^{*}\left(p_{K}\right)⊗a_{l,4x}\left(p_{K}\right)\right]=\left[a_{l,vec}\left(p_{1}\right),\cdots ,a_{l,vec}\left(p_{K}\right)\right]$$

and

(7) $$c={\left[c_{s_{1},4},c_{s_{2},4},\cdots ,c_{s_{K},4}\right]}^{T}$$

Without loss of generality, suppose that the number of sensors of two subarrays of each NA is equal, i.e., $N_{1}=N_{2}=M/2$. Then, the closed-form expression of the $a_{l,vec}\left(p_{k}\right)$ location can be stacked as [40]

(8) $$S_{vec}=\left\{-M_{vec}d,\;-\left(M_{vec}-1\right)d,\;\cdots ,\;\left(M_{vec}-1\right)d,\;M_{vec}d\left|M_{vec}=2N_{1}\left(N_{1}+1\right)-2\right.\right\}$$

with many redundant elements. According to [41], matrix $A_{l,sort}\left(p\right)\in C^{\left(2M_{vec}+1\right)\times K}$ is formed by removing the redundancy from $a_{l,vec}\left(p_{k}\right)$. Meanwhile, the corresponding virtual signal vector $z_{l,sort}$ is given by

(9) $$z_{l,sort}=A_{l,sort}\left(p\right)c$$

Then, according to the geometry relationship showed in Figure 1, for a random grid $p={\left[x,y\right]}^{T}$, we have

(10) $$\sin\varphi_{l}=\frac{x-x_{l}}{\sqrt{{\left(x-x_{l}\right)}^{2}+{\left(y-y_{l}\right)}^{2}}}$$

Let $q_{l}=\frac{J}{2}\sin\varphi_{l}$, where $J=2M_{vec}+1$, yielding

(11) $$q_{l}=\frac{J(x-x_{l})}{2\sqrt{{\left(x-x_{l}\right)}^{2}+{\left(y-y_{l}\right)}^{2}}}$$

Define DFT vector $v_{l,p}\in C^{J\times 1}$, the τth element of which is

(12) $$v_{l,p}\left(\tau \right)={\left(e^{-j2\pi q_{l}/J}\right)}^{\tau }$$

Substitute (11) into (12), yielding

(13) $$v_{l,p}\left(\tau \right)=e^{\frac{-j\pi \tau \left(x-x_{l}\right)}{\sqrt{{\left(x-x_{l}\right)}^{2}+{\left(y-y_{l}\right)}^{2}}}}$$

where 1≤τ≤J. Combining the information of MNAs, we can construct the following DPD problem:

(14) $${\hat{p}}_{k}^{\mathrm{ini}}=\arg\;\max_{p}\sum_{l=1}^{L}\sum_{\tau =1}^{J}v_{l,p}\left(\tau \right)z_{l,sort}\left(\tau \right)$$

where the estimated location ${\hat{p}}_{k}^{\mathrm{ini}}={\left[{\hat{x}}_{k}^{\mathrm{ini}},{\hat{y}}_{k}^{\mathrm{ini}}\right]}^{T}$, k=1,2,⋯,K is based on the assumption that the position of the source falls on the preset grid. When the assumption is not met, the off-grid error will always exist. To overcome this issue, we apply the Taylor compensation method to the initial position estimation.

Define vectors

(15) $$p_{x}={\left[x_{1},x_{2},\cdots ,x_{K}\right]}^{T}$$

(16) $$p_{y}={\left[y_{1},y_{2},\cdots ,y_{K}\right]}^{T}$$

(17) $${\hat{p}}_{x}^{\mathrm{ini}}={\left[{\hat{x}}_{1}^{\mathrm{ini}},{\hat{x}}_{2}^{\mathrm{ini}},\cdots ,{\hat{x}}_{K}^{\mathrm{ini}}\right]}^{T}$$

(18) $${\hat{p}}_{y}^{\mathrm{ini}}={\left[{\hat{y}}_{1}^{\mathrm{ini}},{\hat{y}}_{2}^{\mathrm{ini}},\cdots ,{\hat{y}}_{K}^{\mathrm{ini}}\right]}^{T}$$

(19) $$z_{sort}={\left[z_{1,sort}^{T},z_{2,sort}^{T},\cdots ,z_{L,sort}^{T}\right]}^{T}$$

and matrix

(20) $$A_{sort}\left(p\right)={\left[A_{1,sort}^{T}\left(p\right),A_{2,sort}^{T}\left(p\right),\cdots ,A_{L,sort}^{T}\left(p\right)\right]}^{T}$$

According to (9), we have

(21) $$z_{sort}=A_{sort}\left(p\right)c$$

Performing the first-order Taylor expansion of $A_{sort}\left(p\right)$ at ${\hat{p}}^{\mathrm{ini}}={\left[{\left({\hat{p}}_{1}^{\mathrm{ini}}\right)}^{T},\cdots ,{\left({\hat{p}}_{K}^{\mathrm{ini}}\right)}^{T}\right]}^{T}$, and ignoring the second-order and higher-order terms, we have:

(22) $$A_{sort}\left(p\right)\approx A_{sort}\left({\hat{p}}^{\mathrm{ini}}\right)+\sum_{i=1}^{2K}{\left.\left(p\left(i\right)-{\hat{p}}^{\mathrm{ini}}\left(i\right)\right)\frac{\partial A_{sort}\left(p\right)}{\partial p\left(i\right)}\right|}_{p={\hat{p}}^{\mathrm{ini}}}$$

where $p\left(i\right)$ and ${\hat{p}}^{\mathrm{ini}}\left(i\right)$ extract the *i*th element of p and ${\hat{p}}^{\mathrm{ini}}$, respectively. Substitute (15)–(18) into (22), yielding

(23) $$A_{sort}\left(p\right)\approx A_{sort}\left({\hat{p}}^{\mathrm{ini}}\right)+{\left.\frac{\partial A_{sort}\left(p\right)}{\partial p_{x}^{T}}\right|}_{p_{x}={\hat{p}}_{x}^{\mathrm{ini}}}\Lambda_{x}+{\left.\frac{\partial A_{sort}\left(p\right)}{\partial p_{y}^{T}}\right|}_{p_{y}={\hat{p}}_{y}^{\mathrm{ini}}}\Lambda_{y}$$

where $\Lambda_{x}=\mathrm{diag}\left(\delta_{x}\right)$, $\Lambda_{y}=\mathrm{diag}\left(\delta_{y}\right)$ with $\delta_{x}=p_{x}-{\hat{p}}_{x}^{\mathrm{ini}}$, $\delta_{y}=p_{y}-{\hat{p}}_{y}^{\mathrm{ini}}$. Substitute (23) into (21), yielding

(24) $${\hat{z}}_{sort}\approx \left(A_{sort}\left({\hat{p}}^{\mathrm{ini}}\right)+{\left.\frac{\partial A_{sort}\left(p\right)}{\partial p_{x}^{T}}\right|}_{p_{x}={\hat{p}}_{x}^{\mathrm{ini}}}\Lambda_{x}+{\left.\frac{\partial A_{sort}\left(p\right)}{\partial p_{y}^{T}}\right|}_{p_{y}={\hat{p}}_{y}^{\mathrm{ini}}}\Lambda_{y}\right)c$$

Rewrite (24) as

(25) $${\hat{z}}_{sort}\approx \left[A_{sort}\left({\hat{p}}^{\mathrm{ini}}\right),{\left.\frac{\partial A_{sort}\left(p\right)}{\partial p_{x}^{T}}\right|}_{p_{x}={\hat{p}}_{x}^{\mathrm{ini}}},{\left.\frac{\partial A_{sort}\left(p\right)}{\partial p_{y}^{T}}\right|}_{p_{y}={\hat{p}}_{y}^{\mathrm{ini}}}\right]\left[\begin{array}{c} c\\ \omega_{x}\\ \omega_{y} \end{array}\right]$$

where $\omega_{x}=\Lambda_{x}c$ and $\omega_{y}=\Lambda_{y}c$. Let $B=\left[A_{sort}\left({\hat{p}}^{\mathrm{ini}}\right),{\left.\frac{\partial A_{sort}\left(p\right)}{\partial p_{x}^{T}}\right|}_{p_{x}={\hat{p}}_{x}^{\mathrm{ini}}},{\left.\frac{\partial A_{sort}\left(p\right)}{\partial p_{y}^{T}}\right|}_{p_{y}={\hat{p}}_{y}^{\mathrm{ini}}}\right]$, $y=\left[\begin{array}{c} c\\ \omega_{x}\\ \omega_{y} \end{array}\right]$, the least square solution of y is readily given by

(26) $$\hat{y}={\left(B^{H}B\right)}^{-1}B^{H}{\hat{z}}_{sort}$$

It should be noted that the estimated value $\hat{c}$ is composed of the first to *K*th elements of $\hat{y}$, ${\hat{\omega }}_{x}$ is composed of the $\left(K+1\right)$th to 2Kth elements of $\hat{y}$, and ${\hat{\omega }}_{y}$ is composed of the $\left(2K+1\right)$th to 3Kth elements of $\hat{y}$. Therefore, the accurate position estimation after Taylor compensation is given by

(27) $${\hat{p}}_{x}={\hat{p}}_{x}^{\mathrm{ini}}+{\hat{\omega }}_{x}./\hat{c}$$

(28) $${\hat{p}}_{y}={\hat{p}}_{y}^{\mathrm{ini}}+{\hat{\omega }}_{y}./\hat{c}$$

The main steps of the proposed algorithm are summarized as in Algorithm 1.

**Algorithm** **1:** Main Steps of the FOC-DFT-Taylor Algorithm**Input:** ${\left\{x_{l}\left(t\right)\right\}}_{t=1}^{T}$, *K*, ${\left\{u_{l}\right\}}_{l=1}^{L}$, λ, *d*, *M*, $N_{1}$, $N_{2}$.

Calculate the FOC matrix ${\hat{R}}_{l,4}$ according to (4);Vectorize the matrix ${\hat{R}}_{l,4}$ and remove the redundancy to obtain the virtual signal vector $z_{l,sort}$ utilizing (5) and (8);Construct the normalized DFT vector according to (12) and obtain initial estimated values of the source position ${\hat{p}}_{k}^{\mathrm{ini}}={\left[{\hat{x}}_{k},{\hat{y}}_{k}\right]}^{T},k=1,2,\cdots ,K$ using (14);Perform the first-order Taylor expansion of $A_{sort}\left(p\right)$ at ${\hat{p}}^{\mathrm{ini}}$ and construct the least square constraint utilizing (23) and (26);Obtain accurate position estimation after compensation according to (27) and (28).
**Output:** ${\hat{p}}_{x}$, ${\hat{p}}_{y}$

## 4. Performance Discussion

### 4.1. Complexity

Here, the main computational complexity of different algorithms is compared in terms of complex number multiplication times. The parameters used are listed as follows: *M* is the number of array elements; *K* denotes the number of sources; *L* is the number of NAs; T represents the number of snapshot; $J=2M_{vec}+1$ is the length of virtual signal vector $z_{l,sort}$; and we denote the number of search grids for x and y directions as recorded as $D_{x}$ and $D_{y}$. The main complexity of the proposed algorithm lies in the calculation of the FOC matrix, $O\left(LTM^{4}\right)$; grid search, $O\left(LJD_{x}D_{y}\right)$; complex differentiation, $O\left(2LKJ\right)$; and compensation, $O\left(27K^{3}+9K^{2}+9K^{2}LJ+3KLJ\right)$. For the sake of comparison, the complexity of the proposed algorithm (termed as FOC-DFT-Taylor), the classic SDF DPD algorithm with spatial smoothing technology (termed as FOC-SS-SDF-DPD) [22,42], the Capon DPD algorithm with spatial smoothing technology (termed as FOC-SS-Capon-DPD) [18], and the proposed DFT DPD algorithm with SOC (termed as SOC-DFT-DPD) are given in Table 1, where $N_{a}=\left(J+1\right)/2$ denotes the length of the smoothing window.

**Figure 2:**
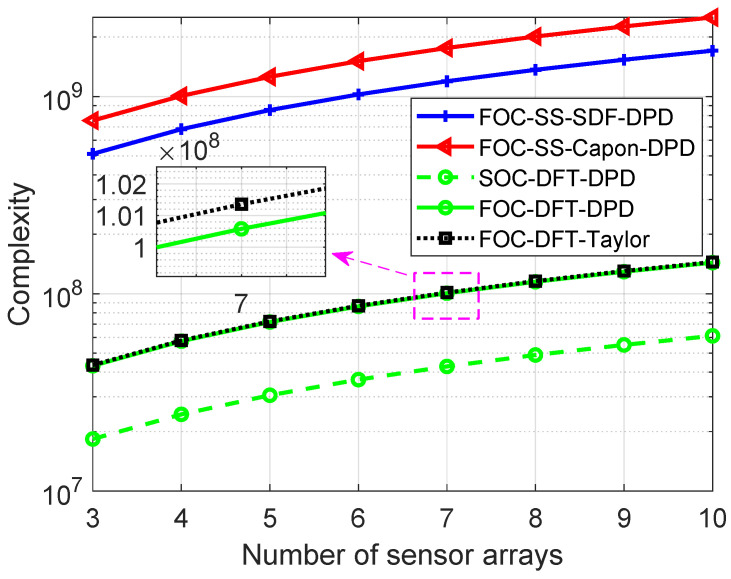
Comparison of complexity versus *L*.

Figure 2 shows the computational complexity of four different algorithms with the parameters setting as below: M=8, K=12, $N_{1}=N_{2}=4$, T=500, J=77, $N_{a}=39$, $D_{x}=D_{y}=400$, and the number of NAs is changed from 3 to 10. As we can see, compared to the FOC-SS-SDF-DPD and FOC-SS-Capon-DPD algorithm, the proposed FOC-DFT-DPD and FOC-DFT-Taylor algorithms reduce the computational complexity thanks to the computationally efficient cost function. The complexity of the proposed FOC-DFT-Taylor is a bit heavier than that of the FOC-DFT-DPD and SOC-DFT-DPD algorithms, while it exhibits higher localization accuracy, which will be shown in Section 5.

**Figure 3:**
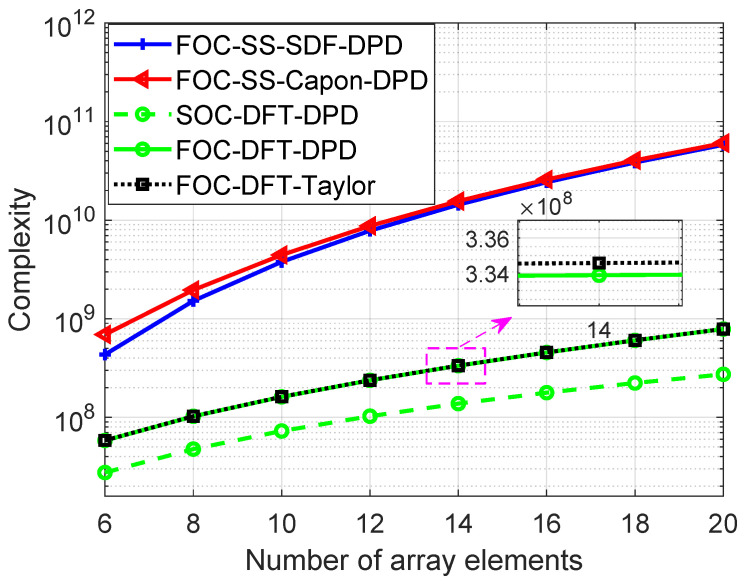
Comparison of complexity versus *M*.

Figure 3 shows the computational complexity of four different algorithms with the parameters setting as below: K=8, $N_{1}=N_{2}=M/2$, T=300, $D_{x}=D_{y}=500$, and the number of array elements is changed from 6 to 20. As we can see, compared to the FOC-SS-SDF-DPD and FOC-SS-Capon-DPD algorithm, the proposed FOC-DFT-DPD and FOC-DFT-Taylor algorithms reduce the computational complexity, as they are less sensitive to the number of array elements *M*. The complexity of the proposed FOC-DFT-Taylor algorithm is a bit heavier than that of FOC-DFT-DPD and SOC-DFT-DPD algorithms.

### 4.2. Advantages

1. *Low Complexity:* Compared to the FOC-SS-SDF-DPD and FOC-SS-Capon-DPD algorithms, the proposed algorithm reduces much of the computational complexity thanks to the computationally efficient cost function.
2. *High DOF:* Compared to the FOC-SS-SDF-DPD and FOC-SS-Capon-DPD algorithms, the proposed algorithm does not need SS technology for decoherence, which is caused by vectorizing the FOC matrix. Thus, the proposed algorithm can estimate more sources.
3. *Suppress Gaussian Noise:* The proposed algorithm takes full advantage of the characteristics of non-Gaussian signals, and the Gaussian noise is suppressed during the process of calculating the FOC matrix.
4. *High Accuracy:* When the sources do not fall on the preset grids, i.e., the off-grid error exists, the proposed algorithm can still estimate the position of sources accurately thanks to the Taylor compensation.
5. *High-Resolution Capability:* Compared to FOC-SS-SDF-DPD and FOC-SS-Capon-DPD methods, the proposed method has higher resolution capability.

## 5. Numerical Analysis

We conduct numerical simulations in this section to evaluate various algorithms and demonstrate the effectiveness of the proposed approach. The superiority of the proposed method is established through comparisons. Our evaluation includes assessing localization accuracy using root mean square error (RMSE) across different algorithms, which is defined as

(29) $$\mathrm{RMSE}=\frac{1}{K}\sum_{k=1}^{K}\sqrt{\sum_{m_{c}=1}^{M_{c}}{∥{\hat{p}}_{k,m_{c}}-p_{k}∥}^{2}/M_{c}}$$

here, $M_{c}$ denotes the number of Monte Carlo runs, and ${\hat{p}}_{k,m_{c}}$ is the estimated position of the *k*th source in the $m_{c}$th Monte Carlo experiment. The resolution capability of the algorithms is assessed by the definition of the effective estimate rate (EER), which is calculated by

(30) $$\mathrm{EER}=\frac{1}{M_{c}}\sum_{m_{c}}^{M_{c}}\alpha_{m_{c}}$$

with

(31) $$\alpha_{m_{c}}=\left\{\begin{array}{c} 1,\;\mathrm{if}\;\frac{1}{K}\sum_{k=1}^{K}∥{\hat{p}}_{k,m_{c}}-p_{k}∥<\varepsilon_{\mathrm{error}}\\ 0,\;\mathrm{otherwise} \end{array}\right.$$

where $\varepsilon_{\mathrm{error}}$ represents the error threshold.

### 5.1. Effectiveness Analysis

**Figure 4:**
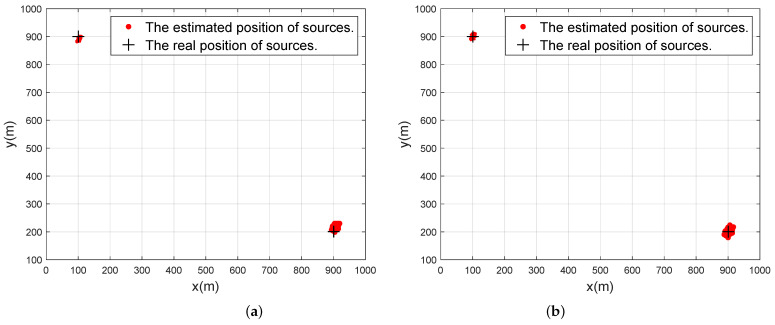
Scatter diagrams of the proposed algorithm when M=4, $N_{1}=N_{2}=2$, T=300, $D_{x}=D_{y}=600$, $u_{1}={\left[-900\;m,1000\;m\right]}^{T}$, $u_{2}={\left[-300\;m,500\;m\right]}^{T}$, $u_{3}={\left[300\;m,-180\;m\right]}^{T}$, $u_{4}={\left[-800\;m,900\;m\right]}^{T}$, $p_{1}={\left[100.5\;m,900.2\;m\right]}^{T}$, $p_{2}={\left[900.6\;m,200.7\;m\right]}^{T}$, SNR=5dB. (**a**) Initial estimation. (**b**) After Taylor compensation.

Figure 4 shows scatter diagrams of the proposed FOC-DFT-Taylor algorithm, where the number of Monte Carlo runs is 300, K=2, and L=4. The location of sources and SAs are set as $u_{1}={\left[-900\;m,1000\;m\right]}^{T}$, $u_{2}={\left[-300\;m,500\;m\right]}^{T}$, $u_{3}={\left[300\;m,-180\;m\right]}^{T}$, $u_{4}={\left[-800\;m,900\;m\right]}^{T}$, $p_{1}={\left[100.5\;m,900.2\;m\right]}^{T}$, $p_{2}={\left[900.6\;m,200.7\;m\right]}^{T}$. The results presented in Figure 4a demonstrate the successful estimation of sources by the FOC-DFT-DPD algorithm. Furthermore, analysis of Figure 4b reveals that the estimated positions closely align with the true positions following Taylor compensation, providing a compelling clue for the efficacy of the proposed FOC-DFT-Taylor algorithm.

### 5.2. RMSE Results

**Figure 5:**
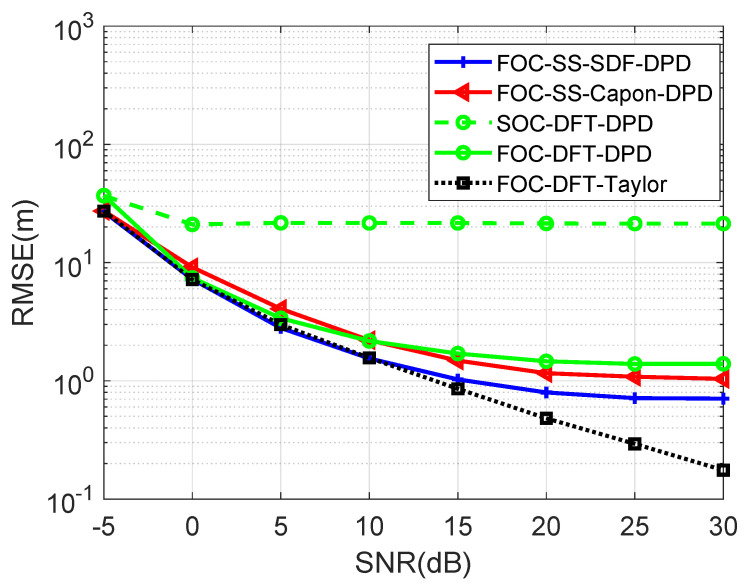
Comparison of RMSE versus SNR.

Figure 5 depicts the RMSE of four algorithms versus SNR when the number of Monte Carlo runs is 600, M=6, $N_{1}=N_{2}=3$, T=500, $D_{x}=D_{y}=600$, L=4, K=2, $u_{1}={\left[-900\;m,-1200\;m\right]}^{T}$, $u_{2}={\left[-300\;m,-1100\;m\right]}^{T}$, $u_{3}={\left[300\;m,-1000\;m\right]}^{T}$, $u_{4}={\left[-800\;m,-900\;m\right]}^{T}$, $p_{1}={\left[200.5\;m,623.5\;m\right]}^{T}$, $p_{2}={\left[920.5\;m,-174.5\;m\right]}^{T}$, SNR is changed from −5dB to 30dB, and the gap of search grids is 1 m. Observe that the RMSE values of the four algorithms exhibit close proximity except for the SOC-DFT-DPD algorithm when SNR is below 10 dB, thanks to the property of non-Gaussian sources. The proposed FOC-DFT-Taylor algorithm demonstrates superior performance over the others at SNR levels exceeding 10 dB, showcasing its exceptional capabilities. Moreover, the RMSE associated with the proposed FOC-DFT-Taylor algorithm consistently remains below that of the FOC-DFT-DPD algorithm at the expense of performing Taylor compensation.

**Figure 6:**
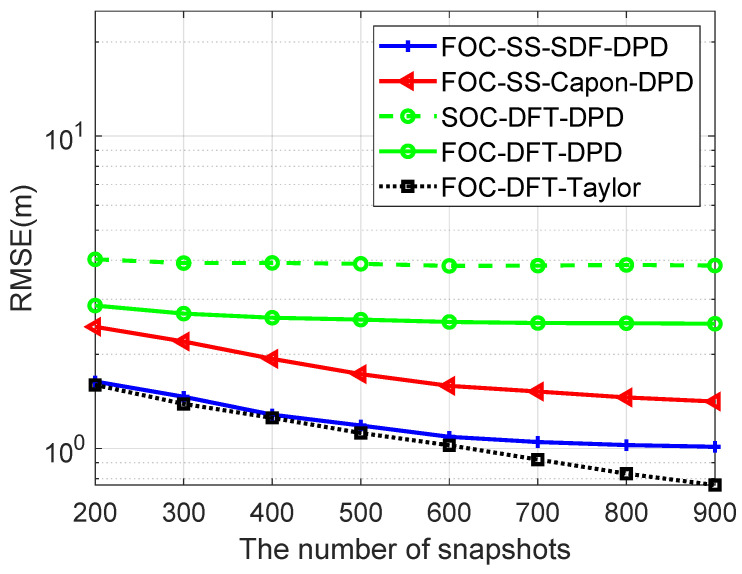
Comparison of RMSE versus T.

Figure 6 shows the RMSE of four algorithms versus T when the number of Monte Carlo runs is 600, M=6, $N_{1}=N_{2}=3$, SNR=15dB, $D_{x}=D_{y}=600$, L=4, K=2, $u_{1}={\left[-900\;m,-1200\;m\right]}^{T}$, $u_{2}={\left[-300\;m,-1100\;m\right]}^{T}$, $u_{3}={\left[300\;m,-1000\;m\right]}^{T}$, $u_{4}={\left[-800\;m,-900\;m\right]}^{T}$, $p_{1}={\left[100.5\;m,815.5\;m\right]}^{T}$, $p_{2}={\left[970.5\;m,54.5\;m\right]}^{T}$, the number of snapshots is changed from 200 to 900, and the gap of the search grids is 1 m. The localization performance of the FOC-DFT-DPD and SOC-DFT-DPD remains consistent, as they do not operate as super resolution algorithms and are constrained by *M*. Thanks to the property of non-Gaussian sources, the localization accuracy of the FOC-DFT-DPD is higher than that of SOC-DFT-DPD. Moreover, the RMSE values of the FOC-DFT-Taylor algorithm consistently outperform the other three algorithms due to its compensation method, showcasing its superior accuracy in localization.

**Figure 7:**
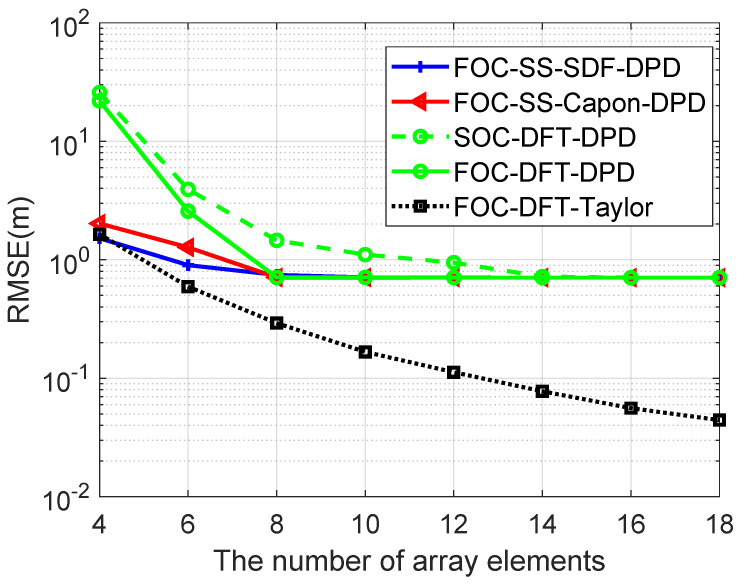
Comparison of RMSE versus *M*.

Figure 7 shows the RMSE performance of different algorithms versus the number of array elements *M* over 500 Monte Carlo runs, when $N_{1}=N_{2}=M/2$, $D_{x}=D_{y}=600$, L=4, K=2, $u_{1}={\left[-900\;m,-1200\;m\right]}^{T}$, $u_{2}={\left[-300\;m,-1100\;m\right]}^{T}$, $u_{3}={\left[300\;m,-1000\;m\right]}^{T}$, $u_{4}={\left[-800\;m,-900\;m\right]}^{T}$, $p_{1}={\left[100.5\;m,815.5\;m\right]}^{T}$, $p_{2}={\left[970.5\;m,54.5\;m\right]}^{T}$, T=500, SNR=20dB, the number of array elements is changed from 4 to 18, and the gap of the search grids is 1 m. Observe that when the array size exceeds 8, the RMSEs of FOC-SS-SDF-DPD, FOC-SS-Capon-DPD, and FOC-DFT-DPD algorithms stabilize around 0.7 m due to their inability to address off-grid errors. In comparison, the RMSE values of the proposed FOC-DFT-Taylor algorithm consistently outperform the other algorithms thanks to its compensation method, demonstrating superior accuracy in localization. Unfortunately, the SOC-DFT-DPD performs poorly, as it fails to take full advantage of non-Gaussian sources. Furthermore, the RMSE values of FOC-DFT-DPD decrease as the array size increases, indicating that localization accuracy is constrained by the number of array elements.

### 5.3. EER Analysis

**Figure 8:**
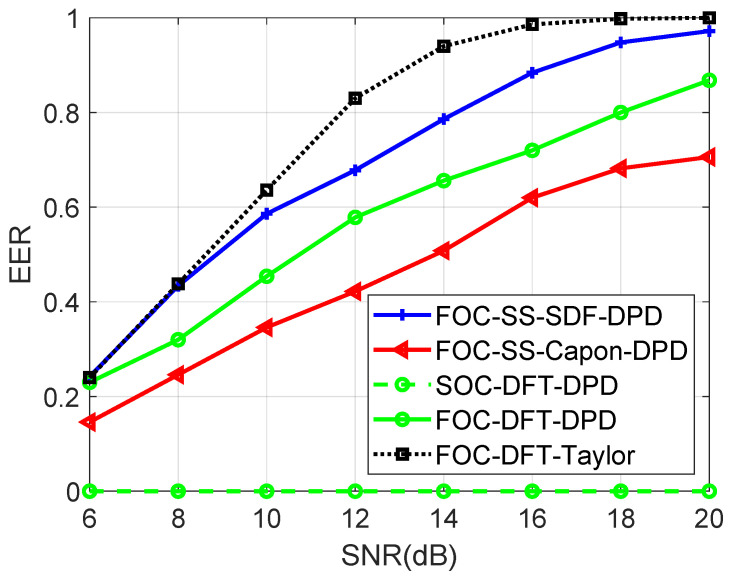
Comparison of EER versus SNR.

Figure 8 shows the EER of four algorithms versus SNR when the number of Monte Carlo runs is 600, M=8, $N_{1}=N_{2}=4$, T=500, $D_{x}=D_{y}=600$, L=4, K=2, $u_{1}={\left[-900\;m,-1200\;m\right]}^{T}$, $u_{2}={\left[-300\;m,-1100\;m\right]}^{T}$, $u_{3}={\left[300\;m,-1000\;m\right]}^{T}$, $u_{4}={\left[-800\;m,-900\;m\right]}^{T}$, $p_{1}={\left[100.5\;m,900.2\;m\right]}^{T}$, $p_{2}={\left[900.6\;m,200.7\;m\right]}^{T}$, SNR is changed from 6 dB to 20 dB, the gap of search grids is 1 m, and the error threshold $\varepsilon_{\mathrm{error}}$ is set as 0.75 m. The EER achieved by the proposed FOC-DFT-Taylor algorithm surpasses 90%, contrasting with the EERs below 90% for the other four algorithms when SNR reaches a level of 14 dB. Unfortunately, the SOC-DFT-DPD performs poorly, as it fails to take full advantage of non-Gaussian sources. Notably, the proposed FOC-DFT-Taylor algorithm exhibits superior resolution capabilities compared to the remaining algorithms in the study.

**Figure 9:**
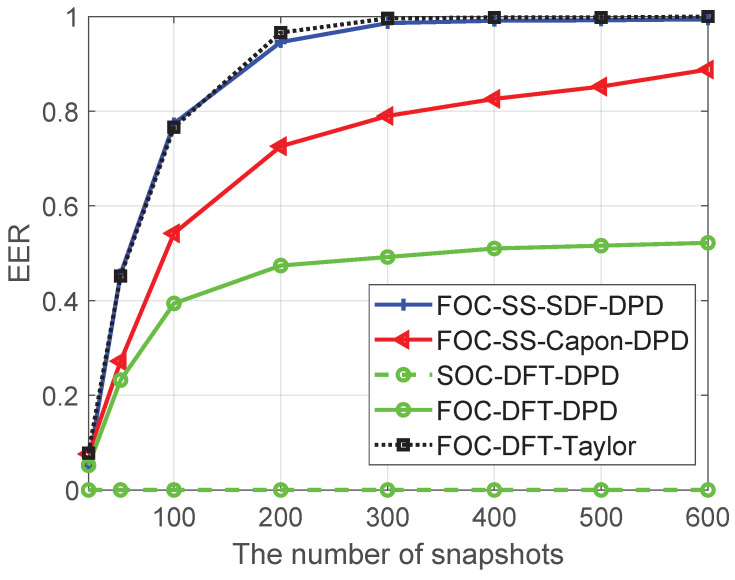
Comparison of EER versus T.

Figure 9 shows the EER of four algorithms versus T, when the number of Monte Carlo runs is 600, M=6, $N_{1}=N_{2}=3$, SNR=20dB, $D_{x}=D_{y}=600$, L=4, K=2, $u_{1}={\left[-900\;m,-1200\;m\right]}^{T}$, $u_{2}={\left[-300\;m,-1100\;m\right]}^{T}$, $u_{3}={\left[300\;m,-1000\;m\right]}^{T}$, $u_{4}={\left[-800\;m,-900\;m\right]}^{T}$, $p_{1}={\left[200.5\;m,623.5\;m\right]}^{T}$, $p_{2}={\left[920.5\;m,-174.5\;m\right]}^{T}$, the number of snapshots is changed from 20 to 600, the gap of search grids is 1 m, and the error threshold $\varepsilon_{\mathrm{error}}$ is set as 1 m. The EER of the proposed FOC-DFT-Taylor algorithm shows a slight increase compared to the FOC-SS-SDF-DPD algorithm, yet with a significant reduction in complexity. Unfortunately, the SOC-DFT-DPD performs poorly, as it fails to take full advantage of non-Gaussian sources. Additionally, when compared to the FOC-SS-Capon-DPD and FOC-DFT-DPD algorithms, the EER of the FOC-DFT-Taylor algorithm is higher, underscoring its superior resolution capabilities.

## 6. Conclusions

In this article, the focus is on discussing the direct position determination (DPD) of non-Gaussian sources and proposing a new algorithm called the FOC-DFT-Taylor algorithm with multiple nested arrays (MNAs). This novel algorithm is designed to create an efficient DPD cost function that significantly reduces complexity when compared to conventional FOC-SS-SDF-DPD and FOC-SS-Capon-DPD algorithms. By leveraging the characteristics of non-Gaussian signals, this proposed algorithm offers a greater number of achievable degrees of freedom (DOFs). Additionally, due to the utilization of Taylor compensation, this new algorithm demonstrates improved localization accuracy and resolution capability over the traditional FOC-SS-SDF-DPD and FOC-SS-Capon-DPD algorithms.

## Figures and Tables

**Table 1:** Comparison of computational complexity.

Methods	Computational Complexity
SOC-DFT-DPD	$O\left(LTM^{2}+LM_{vec}D_{x}D_{y}\right)$
FOC-DFT-DPD	$O\left(LTM^{4}+LJD_{x}D_{y}\right)$
FOC-DFT-Taylor	$O\left(LTM^{4}+LJD_{x}D_{y}+27K^{3}+9K^{2}+9K^{2}LJ+5KLJ\right)$
FOC-SS-SDF-DPD [22]	$O\left(LTM^{4}+2LN_{a}^{3}+LD_{x}D_{y}N_{a}\left(N_{a}-K\right)\right)$
FOC-SS-Capon-DPD [18]	$O\left(LTM^{4}+LN_{a}^{3}+LD_{x}D_{y}\left(N_{a}^{2}+N_{a}\right)\right)$

## Data Availability

The data used to support the findings of this study are included within the article.

## Abbreviations

The following abbreviations are used in this manuscript:

DPD	Direct Position Determination
MNA	Multiple Nested Array
DFT	Discrete Fourier Transform
AOA	Angle of Arrival
TDOA	Time Difference of Arrival
TOA	Time of Arrival
RSS	Received Signal Strength
ML	Maximum Likelihood
SNR	Signal-to-noise Ratio
SDF	Subspace Data Fusion
MUSIC	Multiple Signal Classification
EVD	Eigenvalue Decomposition
OFDM	Orthogonal Frequency Division Multiplexing
SOC	Second-order Cumulant
FOC	Fourth-order Cumulant
ULA	Uniform Linear Array
NA	Nested Array
DOF	Degree of Freedom
SS	Spatial Smoothing
RMSE	Root Mean Square Error
EER	Effective Estimate Rate
